# Facing the future of respiratory virus surveillance: “The mosaic surveillance framework”

**DOI:** 10.1111/irv.13122

**Published:** 2023-03-21

**Authors:** Joshua A. Mott, Isabel Bergeri, Hannah C. Lewis, Anthony W. Mounts, Sylvie C. Briand

**Affiliations:** ^1^ Department of Epidemic and Pandemic Prevention and Preparedness World Health Organization Geneva Switzerland; ^2^ Center for Vaccine Equity The Task Force for Global Health Georgia Decatur USA

**Keywords:** multisource, respiratory viruses, surveillance

## Abstract

It is impossible to address the many complex needs of respiratory virus surveillance with a single system. Therefore, multiple surveillance systems and complementary studies must fit together as tiles in a “mosaic” to provide a complete picture of the risk, transmission, severity, and impact of respiratory viruses of epidemic and pandemic potential. Below we present a framework (WHO Mosaic Respiratory Surveillance Framework) to assist national authorities to identify priority respiratory virus surveillance objectives and the best approaches to meet them; to develop implementation plans according to national context and resources; and to prioritize and target technical assistance and financial investments to meet most pressing needs.

## 1 THE NEED FOR THE MOSAIC FRAMEWORK

As we progress through the coronavirus disease 2019 (COVID‐19) pandemic toward an interpandemic period, countries are faced with the need to sustainably transition their surveillance strategies to monitor influenza, SARS‐CoV‐2, respiratory syncytial virus (RSV), and other respiratory viruses of epidemic and pandemic potential. Population expansion, travel patterns, and global trade also present an ongoing risk of new pandemics and a continuing need to strengthen early warning surveillance. To face these challenges, countries must now increase the number of effective surveillance approaches to address multiple surveillance objectives and extend partnerships for surveillance and collaborative analyses of data across sectors. Doing so will improve data for decision‐making during interpandemic periods and to help ensure that respiratory virus surveillance is both timely and scalable in emergencies.

**FIGURE 1:**
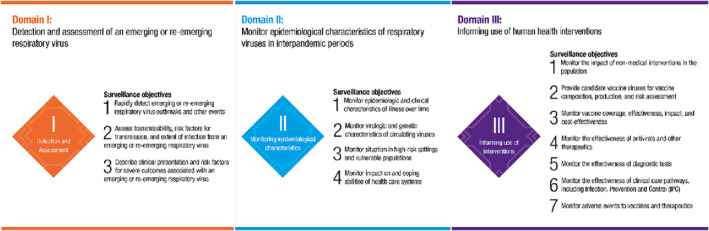
Surveillance domains and associated objectives for respiratory viruses of epidemic and pandemic potential.

WHO Member States have requested a coordinated approach to the sustainable monitoring of respiratory viruses of epidemic and pandemic potential.^1^ For respiratory virus surveillance to function during interpandemic periods and to be resilient to inform decision‐making during times of emergency, surveillance systems must be well‐suited to the objectives that they are being implemented to address. Fit‐for‐purpose surveillance will produce actionable and policy‐relevant information that will engender trust and demonstrate their value for money, increasing commitment by public health authorities to invest in surveillance over time. Conversely, poorly targeted or inefficient surveillance systems may generate sub‐optimal or misleading data for decision‐making, and not be perceived as cost‐effective to sustain or scale in emergencies. Comprehensive surveillance for respiratory viruses involves detecting and assessing emerging or re‐emerging pathogens; monitoring epidemiological and clinical characteristics of illness associated with infections and the virological characteristics of respiratory viruses currently in circulation; and informing the use of human health interventions (Figure 1). As it is impossible to address all these objectives with a single surveillance system, multiple systems and complementary investigations^2^ must work together to address the many information needs of policy makers. In essence, each system or study serves as a tile in a “mosaic”, and only when viewed together will they provide the complete and understandable picture of the human health risk and impact associated with epidemic and pandemic respiratory viruses in circulation.

The COVID‐19 pandemic generated innovations to support surveillance, including those related to improved point of care or self‐test diagnostic technologies, environmental surveillance, community participatory surveillance, and the rapid improvement in global genomic surveillance.^3^ To inform longer‐term surveillance planning, this framework considers some of the benefits, limitations, and most appropriate applications of these innovations to inform possible surveillance strategies.

Critically, national surveillance strategies must be directed by the objectives and information needs of local authorities, locally available resources, and feasibility within the populations under surveillance. Surveillance methods that can help address needed objectives include but are not limited to event‐based surveillance in health‐care facilities, the community, and at the animal–human interface; sentinel surveillance using standardized case definitions and integrated laboratory testing; strong networks of connected public health and clinical laboratories; efficient and comprehensive nationally notifiable disease surveillance systems; targeted surveillance in specific high‐risk settings and vulnerable populations; sustained health‐care capacity monitoring; and enhanced clinical surveillance, among others. Surveillance systems need to be complemented with high‐quality and timely outbreak investigations and studies to obtain information not routinely available from ongoing systems.

**FIGURE 2:**
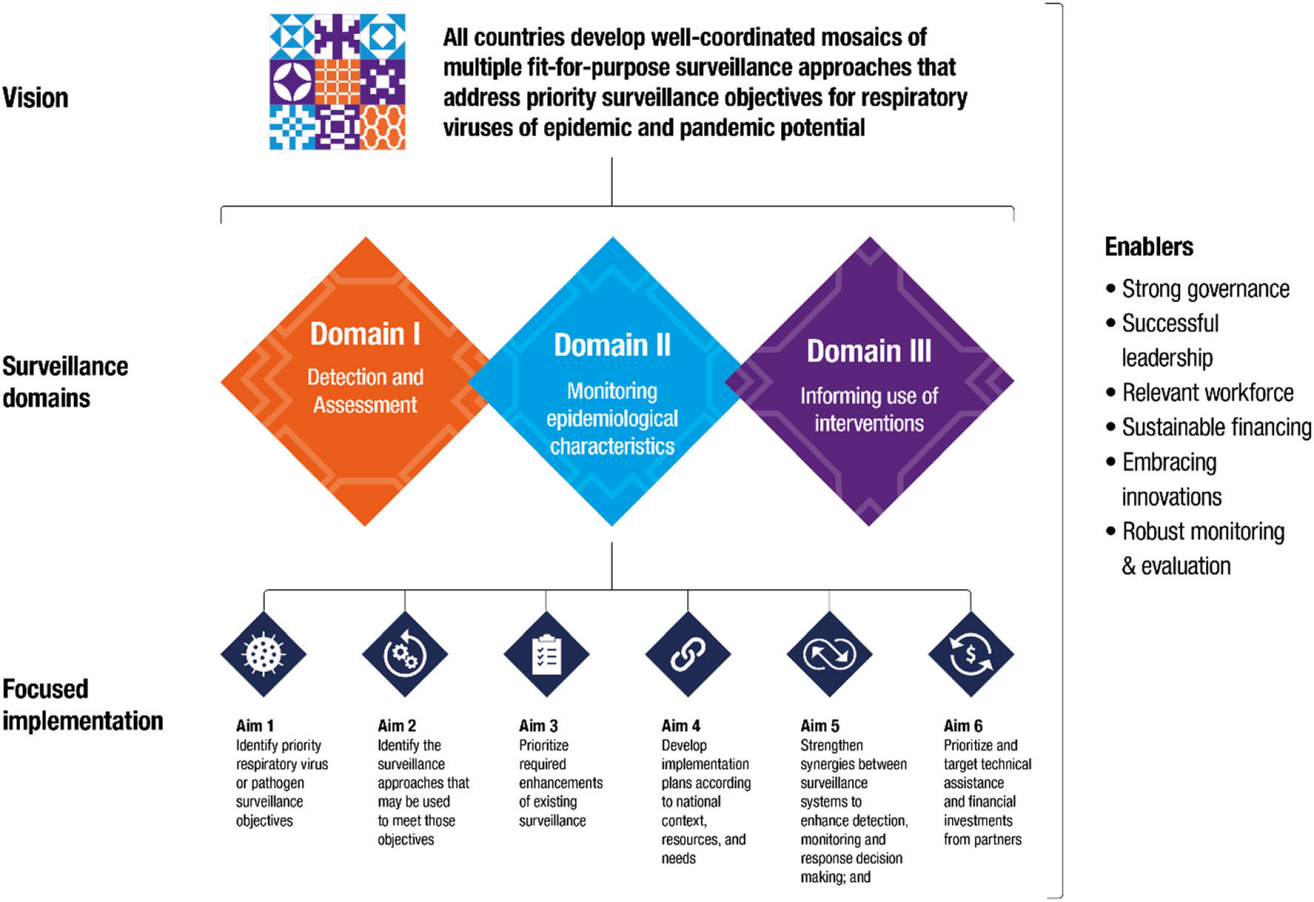
The vision and aims of the mosaic surveillance framework.

There are also several structural or enabling factors that are critical to the success of any sustainable surveillance. These include strong governance and leadership, sustainable financing and workforce, and integration of data standards and appropriate innovations to promote timely and collaborative analyses of surveillance information from multiple sources. Given the complexity of the current respiratory surveillance landscape, national authorities now need an evidence‐based framework to help their countries rapidly (Figure 2):

- identify priority respiratory virus surveillance objectives;
- identify the surveillance approaches that have been used to meet these objectives;
- prioritize required enhancements of existing surveillance;
- develop implementation plans according to the national context, resources, and needs;
- strengthen collaborative synergies between surveillance systems; and
- prioritize and target technical assistance and financial investments from partners

## 2 LEVERAGING GLOBAL EXPERTISE AND EXPERIENCES TO DEVELOP THE MOSAIC FRAMEWORK

The mosaic surveillance framework (which may be accessed at (WHO Mosaic Respiratory Surveillance Framework) was developed by drawing on the experiences of a technical working group (TWG) that included WHO subject matter experts from more than 20 teams/departments at WHO headquarters and members from WHO regional offices (ROs) in all six WHO regions. To further obtain experiences and perspectives from countries of all socio‐economic levels, a secretariat supported WHO ROs as they worked with Country Offices and Ministry of Health partners to implement surveys and gather data on priority objectives, surveillance systems currently used to meet those objectives, and priority surveillance enhancements needed. The ROs also convened regional consultations and focused discussions with countries, informed by surveys, to obtain input for the global consultation. WHO headquarters then hosted a global consultation entitled “’Crafting the Mosaic’: Resilient surveillance systems for respiratory viruses of pandemic potential” on 10 & 11 May 2022. This consultation included 340 in‐person and online attendees including those working in surveillance at the country level, and representatives from WHO Country Offices, all six ROs, WHO headquarters, and external partner organizations. The framework incorporated iterative feedback from the TWG, the WHO COVID‐19 incident management team leadership, and external subject matter experts from the United States Centers for Disease Control and Prevention, the European Centre for Disease Prevention and Control, the Global Fund, the Bill and Melinda Gates Foundation, and the Rockefeller Foundation. The secretariat also collected case studies by working with WHO ROs to obtain examples of implementation of different surveillance systems and studies to meet priority objectives. These case studies provide national authorities with examples of the use of different systems to help them move from a theoretical to practical level.

## 3 COORDINATION WITH EXISTING SURVEILLANCE GUIDANCE AND INITIATIVES

The mosaic surveillance framework is in line with the collaborative surveillance component of the WHO Global Architecture for Health Emergency Preparedness, Response and Resilience (HEPR).^4^ This tool will evolve to ensure it meets countries' needs and synchronizes with the ongoing discussion for the pandemic convention accord + (CA+). This tool also supports the International Health Regulations (IHR) 2005, specifically the core capacity requirements for surveillance and response and the National Action Plans for Health Security,^5^ and will be adapted if the ongoing discussion related to the revision of the IHR (2005) requires it. Importantly, this framework is a conceptual structure that underpins and supports current initiatives and does not supersede any existing global or regional normative surveillance guidance. Rather, it is intended to place the systems represented by existing guidance into a context where they may address the objectives for which they are best intended. The framework presents appropriate uses of existing systems for respiratory virus surveillance and refers to existing global and regional surveillance‐specific guidance and operating procedures wherever they exist.

## 4 IMPLEMENTATION DURING THE COMING YEAR

The specific tools to help countries implement the mosaic surveillance framework will include those to help prioritize respiratory surveillance objectives, map existing systems to priority objectives; and to identify priority enhancements needed. However, associated tools will also help national authorities consider the need to strengthen broader enabling structures for surveillance in their countries.

Our vision is that all countries develop well‐coordinated mosaics of multiple fit‐for‐purpose surveillance approaches that address priority surveillance objectives for influenza, SARS‐CoV‐2, RSV, and other respiratory viruses of epidemic and pandemic potential according to country context. Respiratory viruses serve as a prototypical example of the way surveillance systems can work together resiliently to provide a comprehensive picture of disease emergence, spread, and impact. There are several respiratory viruses that have important commonalities in terms of virological, epidemiological, and clinical surveillance approaches. However, there are areas where this framework has applicability beyond respiratory viruses and, in some cases, beyond respiratory pathogens. The next respiratory pandemic remains imminent, and the time is now to apply lessons learned to assure that newly strengthened and properly focused routine surveillance may better support epidemic, pandemic, and other emergency monitoring needs.

## AUTHOR CONTRIBUTIONS

JAM: Conceptualization, Methodology, Writing, Validation, and Leadership; IB: Conceptualization, Methodology, Writing, Validation, and Leadership; HCL: Methodology, Writing, and Validation; AWM: Writing, Methodology, and Validation; SCB: Conceptualization, Methodology, Validation, and Leadership.

## CONFLICT OF INTEREST STATEMENT

All authors have declared no conflicts of interest.

## DISCLAIMER

More details on methodology and any data to inform the framework may be found at WHO Mosaic Respiratory Surveillance Framework.

### PEER REVIEW

The peer review history for this article is available at https://publons.com/publon/10.1111/irv.13122.

## Data Availability

Data sharing is not applicable ‐ no new data generated.
